# Galangin Attenuates Isoproterenol-Induced Inflammation and Fibrosis in the Cardiac Tissue of Albino Wistar Rats

**DOI:** 10.3389/fphar.2020.585163

**Published:** 2020-11-30

**Authors:** Radhiga Thangaiyan, Sundaresan Arjunan, Kanimozhi Govindasamy, Haseeb A. Khan, Abdullah S. Alhomida, Nagarajan Rajendra Prasad

**Affiliations:** ^1^ Department of Biochemistry and Biotechnology, Faculty of Science, Annamalai University, Tamilnadu, India; ^2^ CAS in Marine Biology, Department of Marine Sciences, Annamalai University, Tamilnadu, India; ^3^ Department of Biochemistry, Dharmapuram Gnanambigai Government Arts College for Women, Tamilnadu, India; ^4^ Department of Biochemistry, College of Science, King Saud University, Riyadh, Saudi Arabia

**Keywords:** galangin, isoproterenol, inflammation, fibrosis, antioxidant, cardiac tissue

## Abstract

Galangin (GA) is an active flavonoid of the rhizome of *Alpinia galanga* that belongs to the ginger family. GA exhibit potent anti-inflammatory properties. Therefore, we evaluated the preventive effects of GA against isoproterenol (ISO)-induced inflammation and myocardial fibrosis in male albino Wistar rats. We found that GA (1 mg/kg b.wt.) pretreatment attenuated the ISO-mediated (5 mg/kg b.wt. for 14 consecutive days) elevation of heart rate, activities of aspartate aminotransferase (AST), alanine aminotransferase (ALT), lactate dehydrogenase (LDH), creatine kinase (CK), creatine kinase-MB (CKMB) in the rat serum. We also noticed that GA prevented the ISO-mediated cardiac markers i.e. cardiac troponin T and I (cTnT and cTnI) expression in the serum of rats. Further, GA pretreatment prevented ISO-mediated lipid peroxidation and diminished blood pressure and loss of antioxidants status in the heart tissue of ISO treated rats. In addition, GA treatment modulates ISO-induced alterations the expressions of tissue inhibitor of metalloproteinases-1 (TIMP-1), p-AKT, glycogen synthase kinase-3β (p-GSK-3β) and peroxisome proliferators-activated receptor-γ (PPAR-γ) in the heart tissue. Furthermore, molecular analysis (PCR array and western blot) revealed that GA pretreatment prevented inflammation and fibrosis related gene expression pattern in ISO-induced rats. Taken together, the results indicate the cardioprotective effect of GA against ISO-induced inflammation and fibrosis. The antioxidant and anti-inflammatory potential of GA could be considered for its cardioprotective effect in the ISO-treated rats.

## Introduction

Cardiovascular disease (CVD) is a most significant cause of demise worldwide. About 17.9 million deaths were recorded in 2016 due to CVD which represents 31% of all global deaths (World Health Organization, 2017). The CVD occurs due to various pathological conditions resulting in morbidity and mortality at high rates (Pallazola et al., 2019). The CVDs includes atherosclerosis, coronary heart disease (CHD), cardiomyopathy, congestive heart failure (CHF) and myocardial infarction (MI) (Benjamin et al., 2019). The MI has been characterized by biochemical and pathological changes with arterial pressure, altered heart rate and ventricular injury (Anderson and Morrow, 2017). The myocardial fibrosis has been considered as an important part of cardiac remodeling. The myocardial fibrosis and MI leads to heart failure and death. The pathogenesis of MI is complex and yet to be understood completely. Several risk factors that include lifestyle, environmental factors, genetic factors, etc. were identified as major risk factors of MI and studying their biochemical and molecular pathogenesis is important for improving prevention strategies (Zhan et al., 2019).

Myocardial fibrosis results from higher myofibroblast activity and disproportionate accumulation of collagen in the myocardial interstitial tissue (Gyöngyösi et al., 2017). Myocardial fibrosis contributes to left ventricular dysfunction in many disorders and predisposes patients to develop heart failure. Further, during MI there was a deposition of excess fibrous tissue (i.e., collagen types I and III fibers) within the myocardial interstitial tissue (Cowling et al., 2019). Matrix metalloproteinases (MMPs) play a critical role in the heart tissue matrix remodeling (Thenappan et al., 2018). Further, oxidative stress and mitochondrial dysfunction contributes for the pathogenesis of MI. Several authors reported the involvement of mitochondria in the regulation of calcium concentration (Ca^2+^) in cardiac myocytes. The calcium ions regulate mitochondrial ATP production and contractile activity and thus play a pivotal role in the function of cardiac muscle (Upadhyay, 2018). The L-type Ca^2+^ channel has been reported to regulate mitochondrial function. The L-type Ca^2+^ channel kinetics are altered in cTnI-G203S cardiac myocytes and that causes an alteration in mitochondrial membrane potential and metabolic activity of cTnI-G203S cardiac myocytes (Viola and Hool, 2017).

Recently several experimental methods and models were developed to evaluate the preventive effect of pharmaceuticals against MI and heart failure. The isoproterenol hydrochloride (ISO), a *ß*-adrenergic agonist, induces oxidative stress-mediated myocardial infarction in experimental models. ISO has emerged as a pivotal mediator of collagen fragment synthesis in failing hearts, which is accompanied by increased MMPs expression (Radhiga et al., 2019). Therefore, researchers widely employ ISO-experimental animals for the studies of pharmacological intervention. Several molecular pathways are activated during ISO-induced MI which was considered to be the major targets for the drug discovery aspects. The activation of transforming growth factor-β (TGF-β) causes the proliferation of myocardial fibroblasts that induces the synthesis of extracellular matrix proteins (Yousefi et al., 2020). Further, the TGF-β induces inflammatory signaling through activation of serine/threonine kinase receptors, TGF-β RI and II. These activated receptors, in turn, phosphorylate Smad2 and Smad3 and form a Smad complex with Smad4 which translocate into the nucleus (Hata and Chen, 2016). The *a*-Smooth muscle actin (α-SMA) caused fibrogenesis and produced scar tissue during MI.

Several intracellular signaling cascade events were activated during MI. There was a significant activation of mitogen activated kinase (MAPK) three subunits-extracellular regulated kinase ½ (ERK1/2), c-Jun-N-terminal kinase (JNK) and p38. Unregulated expression of JNK and p38 activates pro-inflammatory pathways in the heart tissue (Yokota and Wang, 2016). The MAPK signaling elements also interact transcription factors like nuclear factor-κB (NF-κB) which further stimulates the production of tumor necrosis actor-α (TNF-α), interleukin-1β (IL-β), IL-6, IL-10 and IL-18 etc. Various studies have reported that ISO-treatment mimics clinical MI that induces TNF-α and IL-6 activation thus contributing to inflammation in response to myocardial injury (Boarescu et al., 2019).

Over the past decade, several synthetic drugs have been developed for treatment of the cardiovascular disorders. The synthetic drugs including statins and *ß*-blockers show undesirable adverse effects in the CVD clinics. The cardiovascular drug market is severely impacted by the scarcity of new potential alternative drugs (Shaito et al., 2020). Herbal medicines, in addition to their traditional values, also hold great public and medical interest worldwide as sources of novel lead compounds for cardiovascular drug development (Liperoti et al., 2017). Natural medicinal compounds gain increasing attraction in the treatment of CVDs and in the understanding of disease mechanisms. Several natural compounds have mechanistically been proved as potential agents against cardiac toxicity and MI. Galangin (GA, 3, 5, 7-trihydroxyflavone) is the active flavonoid of the rhizome of *Alpinia galanga*, a plant belongs to the ginger family (Zingiberaceae). GA is a potent scavenger of free radicals and it shows a strong anti-inflammatory property in several experimental models (Cao et al., 2016; Aladaileh et al., 2019). Yang et al. reported that GA modulates thrombin-induced MMP-9 activation in neuroblastoma cell lines via inhibiting protein kinase-dependent NF-κB activation (Yang et al., 2018). Further, GA protects rheumatoid arthritis fibroblast-like synoviocytes via modulation of NF-κB/NLRP3 signaling pathway (Fu et al., 2018). Chen et al. showed that GA inhibited LPS-induced expression of TNF-α, IL-6, IL-1β, COX-2, and iNOS via phosphorylation of JNK, p38, protein kinase B (AKT), and NF-κB p65 (Chen et al., 2018). Therefore, it is postulated that GA could able to modulate the expression of pro-inflammatory molecules. However, GA ability against ISO-induced inflammation and fibrosis in rat myocardial tissue has not been sufficiently investigated. Hence, we investigated the protective effect of GA against ISO-mediated oxidative stress and subsequent inflammation and fibrosis in the cardiac tissue.

## Materials and Methods

### Chemicals

Isoproterenol hydrochloride (ISO) and galangin (GA) were purchased from Sigma-Aldrich, United States. Nitrocellulose membrane, Western ECL Substrate kit were purchased from Bio-Rad, Germany. TNF-α, NF-κB, IL-6, COX-2, iNOS, MMP-2, MMP-9, TGF-β, Fibronectin, *a*-SMA, collagen-I and *ß*-actin antibodies were procured from Santa-Cruz Biotechnology, USA and Goat anti-rabbit, anti-mouse and rabbit anti-goat secondary antibodies were purchased from Sigma-Aldrich, United States. All other fine chemicals and consumables obtained from HiMedia, India.

### Experimental Animals

The experiments were conducted in male albino Wistar rats (weighing 160–180 g, aged 5–7 weeks) which were obtained from Biogen Laboratory Animal Facility, Bangalore. The animals were maintained at 25 ± 3°C with a light/dark cycle. Animal handling and experimental procedures were approved by the Institutional Animal Ethics Committee, Annamalai University and animals were cared in accordance with the “Guide for the Care and Use of Laboratory Animals” and “Committee for the Purpose of Control and Supervision on Experimental Animals” (Qadri and Ramachandra, 2018). The study plan and methods were approved by the Institutional Animal Ethics Committee of Rajah Muthiah Medical College and Hospital (Reg No.160/1999/CPCSEA, Proposal number: 1130), Annamalainagar.

### Experimental Induction of Myocardial Infarction and Dose Determination Study

**FIGURE 1:**
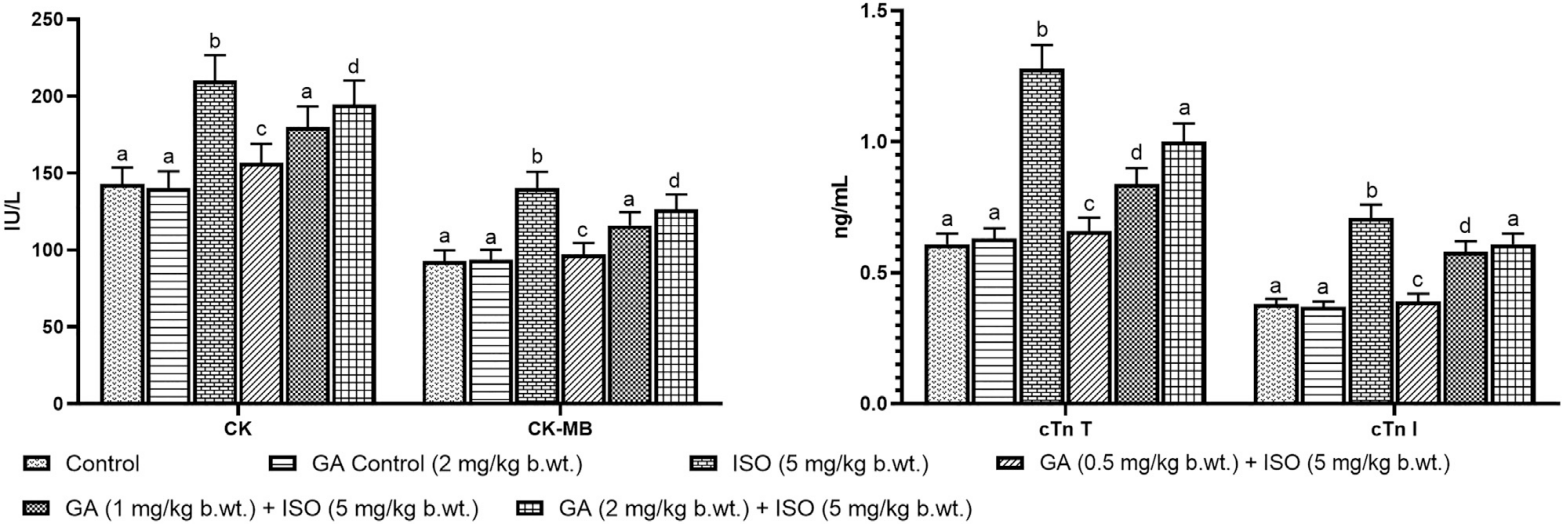
Effect of GA on cardiac markers levels such as CK, CK-MB, cTnT and cTnI in the serum of control and ISO-induced rats. **(A)** Effect of GA on CK and CK-MB in the serum of control and ISO-induced rats. **(B)** Effect of GA on cTnT and cTnI in the serum of control and ISO-induced rats. Values are given as means ± SD of six experiments in each group. Values not sharing a common superscript (a,b,c..) differ significantly at *p* ≤ 0.05 (DMRT).

Myocardial infarction was experimentally induced by subcutaneous (s.c.) injection of ISO (5 mg/kg b.wt.) for 14 days as described as earlier report Maulik et al., 2012). A preliminary study was carried out to determine the optimum dose of GA by assessing serum enzyme activities in ISO-induced rats. GA was given at different doses (i.e. 0.5, 1 and 2 mg/kg b.wt.) to different groups of animals. GA was dissolved in corn oil and given orally once in a day for 14 days. Among the three doses the 1 mg/kg b.wt. dose was more effective other than two doses. Therefore, 1 mg/kg/b.wt. of GA was used for further study (Figures 1A,B).

### Study Design

Group I The rats were divided into four experimental groups with six rats each (*n* = 6).
Group II Control rats received standard pellet diet.
Group III Oral administration of GA (1 g/kg b.wt.) for 14 days using an intragastric tube.
Group IV ISO (5 mg/kg b.wt.) by *s.c.* injection for 14 days.
Group V Oral administration of GA (1 mg/kg b.wt) using an intragastric tube before each ISO (5 mg/kg b.wt.) injection for 14 ays.

After the experimental period, the experimental rats were anesthetized by ketamine hydrochloride (24 mg/kg b.wt.) and sacrificed. Blood samples were collected and serum was separated by centrifugation at 600 *g* for 10 min. The separated serum was used for the analysis of serum marker enzymes. Blood samples were centrifuged and the plasma was separated by aspiration. The separated plasma was used for the analysis of lipid peroxidative markers and non-enzymatic antioxidants. After separation of plasma the buffy coat white cells was removed and the remaining erythrocytes were washed with saline. The erythrocyte was separated by centrifugation at 800 *g* for 10 min and the supernatant was used for the estimation of enzymatic antioxidants. The heart tissue was excised immediately after cervical dislocation and used for histological analysis, biochemical estimations and western blot analysis.

### Measurement of Blood Pressure by Non-Invasive Method

Systolic and diastolic blood pressure and heart rate were measured initial and final day of the experimental period of the study by tail-cuff method (IITC, model 31, United States). Values reported are the average of sequential blood pressure measurements. All the recordings and data analyses were done using a computerized data acquisition system and software.

### Activities of Serum Marker Enzymes

The enzymes like AST, ALT and LDH were analyzed by the method of Reitman and Frankel, (1957), Kim et al, (2008), King and Waind, (1960). The activities of CK and CK-MB was analyzed by a commercial kit manufactured by Agappe Diagnostics Ltd, India. Serum cTn T and I were estimated using enzyme immunoassay commercial kits (Larue et al., 1993).

### Measurement of Lipid Peroxidation and Antioxidants Status

Heart tissue (250 mg) was sliced into pieces and homogenized in an appropriate buffer in cold condition (pH 7.0) to give 10% homogenate (w/v). The homogenates were centrifuged at 200 g for 10 min at 0°C in cold centrifuge. The supernatant was used for the estimation of lipid peroxidation and the analysis of antioxidants status. Lipid peroxidation level was analyzed by estimating thiobarbituric acid reactive substances (TBARS) (Zeb and Ullah, 2016). Lipid hydroperoxides (LHP) level was analyzed by estimating butylated hydroxytoluene (BHT)-reactive substance as per the method of Jiang et al. (1992) *.*

Superoxide dismutase (SOD) activity was estimated by the method of Kakkar et al. (1984). Catalase (CAT) activity was assayed by the procedure of Hadwan (2016). The activity of glutathione peroxidase (GPX) was assayed by the method of Chow and Tappel (1974). Reduced glutathione (GSH) was estimated by the method of Ellman (1959) and GSSG (Tietze, 1969). Vitamin C was estimated by the method of Roe and Kuether (1943), Vitamin E was estimated by the method described by Baker et al. (1981).

### Inflammatory Signaling and Cardiac Fibrotic Gene Expression by PCR Array

The total RNA content was isolated from control, GA control, ISO and GA + ISO treated tissues using an RNeasy mini kit (Qiagen, India) and used for qRT-PCR arrays. The purity of the isolated RNA was measured by Nanodrop spectrophotometer. The cDNA was reverse transcribed using a First Strand cDNA Synthesis Kit and used for PCR amplification by SYBR green chemistry using the Qiagen kit. The relative expression pattern of inflammatory genes (TNF-α, IL-6 IL-10, IL-18, Interferon gamma (IFNγ), IL-1β, COX-2, NF-κB, inhibitor of kappa B-α (IκB-α), iNOS, signal transducer and activator of transcription-3 (STAT-3), cTnT and cTnI) and fibrotic genes (MMP-2, MMP-9, TGF-β1, Fibronectin, *a*-SMA, Collagen-I, Collagen-III, Smad-2, Smad-3, TIMP-2, TIMP-1, Angiotension II receptor, Connective tissue growth factor (CTGF), Endothelin-1 (ET-1), Activator protein (AP-1), intercellular cell adhesion molecule-1 (ICAM-1), vascular cell adhesion molecule-1 (VCAM-I), E-selectin, p-AKT, p38, JNK, ERK, Glycogen synthase kinase 3 (p-GSK-3β), *ß*-catenin, Peroxisome proliferators-activated receptor-γ (PPAR-γ) and myocardin-related transcription factor (MRTF) were analyzed by a PCR array. The fold changes of gene expression were plotted as clustergram. Relative gene expression (RQ) was calculated using 2^−ΔΔCt^.

### Western Blot Analysis for Inflammatory and Cardiac Fibrotic Protein Expression

The heart tissue was homogenized in an ice-cold RIPA buffer containing a protease inhibitor cocktail. The cardiac tissue homogenate was centrifuged at 11,000 g for 10 min at 4°C to remove debris. The supernatant was used to determine the protein concentration by the method of Lowry et al. (1951). Proteins were separated by 10% sodium dodecyl sulfate–polyacrylamide gel electrophoresis (SDS-PAGE) and transferred to nitrocellulose membrane using a Bio-Rad semi-dry western blotting system (Thangaiyan et al., 2018). Membranes were blocked by tris-buffered saline (TBS) containing 5% bovine serum albumin (BSA) for 2 h at room temperature. The membranes were incubated with primary antibodies (TNF-α, NF-κB, IL-6, COX-2, iNOS, MMP-2, MMP-9, TGF-β1, Fibronectin, *a*-SMA, collagen-I and *ß*-actin) in the above solution on an orbit shaker at 4°C for 12 h. Membranes were washed with TBS containing 0.1% Tween 20 solution and incubated with horseradish peroxidase (HRP)-conjugated secondary antibodies for 2 h. The nitrocellulose membranes were washed again with TBST solution and the developed bands were detected using a chemiluminescence substrate kit. The images were acquired and analyzed by Image Studio software (LI-COR).

**Histopathology of Heart Tissue – Hematoxylin & Eosin Staining**

The heart (left ventricle) tissues fixed in 10% formalin after dissection. The sections in the slides were incubated in an oven at 60°C for 30 min then immersed in xylene for 5 min and 4 min; and subsequently immersed in absolute ethanol for 3 and 2 min respectively. The slides were kept in hematoxylin for 7 min, and then water bath for 2–3 min. After dehydration with 70% ethanol for 1min, slides were stained with the working eosin solution for 11 min. The slides were dehydrated with 95% ethanol for 30 s and washed three times with 100% ethanol for 30 s and mounted in a neutral deparaffinated xylene (DPX) medium using standard protocols.

### Massion’s Trichrome and Picrosirius Red Staining

The sections in the slides were incubated in an oven at 60°C for 30 min then immersed in xylene for 5 min, 4 min and subsequently in absolute ethanol for 3 min, 2 min respectively. Sections were brought into water and then stained the nuclei with an acid resistant nuclear stain. After washing well with water; the slides were placed into Massion’s trichrome and picrosirius red solution for 90 min. The sections in the slides were differentiated with 0.01M HCl for 2 min. The slides were placed in 70% ethanol for 30 s, 90% ethanol for 30 s and 100% ethanol for 30 s and repeated three changes and then mounted using neutral deparaffinated xylene (DPX). All the slides were assessed using light microscopy by a pathologist and photographed.

### Statistical Analysis

All the experimental values of this study were expressed as means ± S.D. The data were analyzed by one-way analysis of variance (ANOVA) for the comparison between the groups and the group means were analyzed by Duncan’s multiple range test (DMRT) using SPSS (Version17.0). Microsoft word and Excel 2010 was used for the manuscript preparation statistical and graphical evaluations. *p*-value is the ≤0.05 were considered as statistically significant.

## Results

### Effect of GA on Isoproterenol-Induced Blood Pressure and Heart Rate Measurement

**FIGURE 2:**
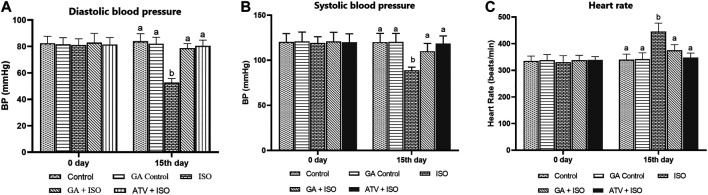
Effect of GA on blood pressure and heart rate in control and ISO-induced rats. **(A)** Effect of GA on diastolic blood pressure measurement in control and ISO-induced in rats **(B)** Effect of GA on systolic blood pressure measurement in control and ISO-induced in rats. **(C)** Effect of GA on heart rate measurement in control and ISO-induced rats. Values are given as means ± SD of six experiments in each group. Values not sharing a common superscript (a,b,c..) differ significantly at *p* ≤ 0.05 (DMRT).

Effect of GA on systolic and diastolic blood pressure and heart rate were shown in Figures 2A,B,C respectively. ISO-induced rats (group III) showed significantly (*p* < 0.05) increase in heart rate and decreased blood pressure when compared to control rats. Pretreatment with GA and ATV reduced heart rate and normalized the blood pressure in ISO-induced rats.

### Effect of GA on ISO-Induced of Serum Marker Enzymes

ISO-treated rats (Group III) showed a significant (*p < 0.05*) increase in the activities of serum marker enzymes such as AST, ALT, LDH, CK, CK-MB and the levels of cTnT and cTnI when compared to control rats (Table 1). Pretreatment with GA (Group IV)/ATV (group V) significantly (*p < 0.05*) prevented ISO-induced activities of AST, ALT, LDH, CK and CK-MB and the levels of cTnT and cTnI in the blood stream.

**TABLE 1:** Effect of GA on AST, ALT, LDH, CK, CK-MB, cTnT and cTnI in the serum of control and ISO-induced rats.

Groups	AST (IU/L)	ALT (IU/L)	LDH (IU/L)	CK (IU/L)	CK-MB (IU/L)	cTnT (ng/ml)	cTnI (ng/ml)
Control	74.62 ± 9.08^a^	22.45 ± 3.47^a^	220.07 ± 20.57^a^	145.17 ± 19.18^a^	94.58 ± 4.28^a^	0.62 ± 0.04^a^	0.35 ± 0.03^a^
GA control (1 mg/kg b.wt.)	73.67 ± 8.15^a^	21.57 ± 2.81^a^	218.43 ± 22.82^a^	142.57 ± 13.19^a^	92.92 ± 3.62^a^	0.61 ± 0.07^a^	0.34 ± 0.02^a^
ISO (5 mg/kg b.wt.)	110.62 ± 10.24^b^	52.52 ± 6.07^b^	280.15 ± 29.27^b^	215.97 ± 19.81^b^	135.36 ± 09.52^b^	1.25 ± 0.13^b^	0.68 ± 0.05^b^
GA (1 mg/kg b.wt.) + ISO (5 mg/kg b.wt.)	77.48 ± 10.90^a^	25.54 ± 3.57^a^	224.27 ± 23.35^a^	151.17 ± 14.25^a^	98.69 ± 4.62^a^	0.65 ± 0.07^a^	0.38 ± 0.02^a^
ATV (1 mg/kg b.wt.) + ISO (5 mg/kg b.wt.)	75.18 ± 8.35^a^	23.28 ± 3.18^a^	221.71 ± 29.04^a^	148.38 ± 15.36^a^	93.27 ± 6.41^a^	0.63 ± 0.10^a^	0.36 ± 0.03^a^

Values are expressed as means ± S.D for six rats in each group. Values not sharing a common superscript. ^(a,b,c..)^ differ significantly at *p* ≤ 0.05 (DMRT).

### Effect of GA on ISO-Induced Oxidative Damages

Lipid peroxidation is the major event of ISO-induced cardiotoxicity. In this study, ISO-treatment (Group III) increased the levels of TBARS and LHP in plasma and heart tissue homogenate. Whereas, pretreatment of GA (Group IV)/ATV (group V) considerably prevented ISO-induced lipid peroxidation (Table 2). The enzymatic and non-enzymatic antioxidants protect the heart tissues against oxidative damages. The plasma and heart tissue of non-enzymatic antioxidants like vitamin C, vitamin E, and GSH were found to be significantly (*p < 0.05*) decreased in ISO-induced rats (Group III) when compared to control rats. However, pretreatment with GA (Group IV)/ATV (group V) significantly (*p < 0.05*) prevented the level of non-enzymatic antioxidants in the plasma and heart tissues in ISO-induced rats (Table 3).

**TABLE 2:** Effect of GA on the levels of TBARS and LHP in the plasma and heart of control and ISO-induced rats.

Groups	Control	GA control (1 mg/kg b.wt.)	ISO (5 mg/kg b.wt.)	GA (1 mg/kg b.wt.) + ISO (5 mg/kg b.wt.)	ATV (1 mg/kg b.wt.) + ISO (5 mg/kg b.wt.)
Plasma (mmol/dl)					
TBARS	0.18 ± 0.01^a^	0.16 ± 0.01^a^	0.45 ± 0.05^b^	0.20 ± 0.02^a^	0.17 ± 0.02^a^
LHP	8.21 ± 0.72^a^	8.19 ± 0.85^a^	19.08 ± 1.81^b^	9.29 ± 1.04^a^	8.41 ± 0.86^a^
Heart (mmol/100 g wet tissue)					
TBARS	0.58 ± 0.05^a^	0.54 ± 0.04^a^	1.65 ± 0.18^b^	0.62 ± 0.04^a^	0.56 ± 0.05^a^
LHP	68.24 ± 4.17^a^	67.28 ± 5.36^a^	112.35 ± 10.08^b^	72.54 ± 5.28^a^	69.19 ± 5.37^a^

Values are expressed as means ± S.D for six rats in each group. Values not sharing a common superscript. ^(a,b,c..)^ differ significantly at *p* ≤ 0.05 (DMRT).

**TABLE 3:** Effect of GA on the levels of vitamin C, vitamin E and GSH in the plasma and heart of control and ISO-induced rats.

Groups	Control	GA control (1 mg/kg b.wt.)	ISO (5 mg/kg b.wt.)	GA (1 mg/kg b.wt.) + ISO (5 mg/kg b.wt.)	ATV (1 mg/kg b.wt.) + ISO (5 mg/kg b.wt
Plasma (mg/dl)					
Vitamin C	2.54 ± 0.24^a^	2.53 ± 0.20^a^	1.52 ± 0.12^b^	2.60 ± 0.30^a^	2.55 ± 0.22^a^
Vitamin E	1.95 ± 0.12^a^	1.96 ± 0.15^a^	1.06 ± 0.18^b^	1.90 ± 0.18^a^	1.93 ± 0.16^a^
GSH	34.20 ± 3.04^a^	35.28 ± 2.58^a^	20.18 ± 1.83^b^	31.27 ± 2.85^a^	33.92 ± 2.52^a^
GSSG	15.86 ± 1.15^a^	15.47 ± 1.48^a^	8.37 ± 0.74^b^	13.95 ± 1.28^a^	14.27 ± 1.12^a^
GSH/GSSG	32.20 ± 3.38^a^	32.28 ± 2.85^a^	18.18 ± 1.68^b^	29.27 ± 2.28^c^	31.92 ± 2.78^a^
Heart (µg/mg protein)					
Vitamin C	0.58 ± 0.07^a^	0.56 ± 0.04^a^	0.30 ± 0.02^b^	0.54 ± 0.04^a^	0.55 ± 0.06^a^
Vitamin E	2.88 ± 0.19^a^	2.90 ± 0.20^a^	1.75 ± 0.14^b^	2.84 ± 0.22^a^	2.86 ± 0.21^a^
GSH	8.65 ± 0.72^a^	8.66 ± 0.72^a^	5.02 ± 0.45^b^	8.60 ± 0.70^a^	8.63 ± 0.78^a^
GSSG	4.38 ± 0.58^a^	4.41 ± 0.39^a^	1.79 ± 0.19^b^	4.29 ± 0.36^a^	4.34 ± 0.41^a^
GSH/GSSG	6.65 ± 0.42^a^	6.66 ± 0.57^a^	3.02 ± 0.29^b^	6.60 ± 0.65^a^	6.63 ± 0.61^a^

Values are expressed as means ± S.D for six rats in each group. Values not sharing a common superscript.^(a,b,c..)^ differ significantly at *p* ≤ 0.05 (DMRT).

The enzymatic antioxidant recycling mechanism preserves cardiac tissue homeostasis. The activities of major enzymatic antioxidants like SOD, CAT, and GPx were found to be (*p < 0.05*) decreased in the erythrocytes and cardiac tissue of ISO-treated rats (Group III). Whereas, pretreatment with GA (Group IV)/ATV (group V) significantly (*p < 0.05*) prevented ISO-induced loss of enzymatic antioxidants status in erythrocytes and heart tissue of rats (Tables 4, 5).

**TABLE 4:** Effect of GA on the activities of SOD, CAT and GPx in the erythrocytes of control and ISO-induced rats.

Groups	SOD (U*/mg of Hb)	CAT (U**/mg of Hb)	GPx (U^@^/mg of Hb)
Control	7.20 ± 0.68^a^	188.69 ± 12.85^a^	13.72 ± 1.05^a^
GA control (1 mg/kg b.wt.)	7.22 ± 0.72^a^	189.62 ± 19.08^a^	13.91 ± 1.21^a^
ISO (5 mg/kg b.wt.)	5.18 ± 0.41^b^	140.29 ± 10.17^b^	10.08 ± 0.92^b^
GA (1 mg/kg b.wt.) + ISO (5 mg/kg b.wt.)	7.12 ± 0.60^a^	178.60 ± 12.51^a^	13.48 ± 1.38^a^
ATV (1 mg/kg b.wt.) + ISO (5 mg/kg b.wt.)	7.21 ± 0.58^a^	186.92 ± 19.51^a^	13.86 ± 1.23^a^

Values are expressed as means ± S.D for six rats in each group. Values not sharing a common superscript. ^(a,b,c..)^ differ significantly at *p* ≤ 0.05 (DMRT). U*, Enzyme concentration required to inhibit the chromogen produced by 50% in 1 min under standard condition; U**, µmole of hydrogen peroxide decomposed/min; U^@^, µmole of GSH utilized/min.

**TABLE 5:** Effect of GA on the activities of SOD, CAT and GPx in the heart of control and ISO-induced rats.

Groups	SOD (U*/mg protein)	CAT (U**/mg protein)	GPx (U^@^/mg protein)
Control	7.50 ± 0.71^a^	53.68 ± 6.28^a^	6.10 ± 0.57^a^
GA control (1 mg/kg b.wt.)	7.55 ± 0.68^a^	54.54 ± 7.29^a^	6.21 ± 0.74^a^
ISO (5 mg/kg b.wt.)	5.08 ± 0.59^b^	30.57 ± 4.69^b^	4.18 ± 0.63^b^
GA (1 mg/kg b.wt.) + ISO (5 mg/kg b.wt.)	7.45 ± 0.76^a^	50.64 ± 4.61^a^	6.00 ± 0.68^a^
ATV (1 mg/kg b.wt.) + ISO (5 mg/kg b.wt.)	7.53 ± 0.98^a^	52.40 ± 6.18^a^	6.18 ± 0.77^a^

Values are expressed as means ± S.D for six rats in each group. Values not sharing a common superscript ^(a,b,c..)^ differ significantly at *p* ≤ 0.05 (DMRT). U*, Enzyme concentration required to inhibit the chromogen produced by 50% in 1 min under standard condition; U**, µmole of hydrogen peroxide decomposed/min; U^@^, µmole of GSH utilized/min.

### Effect of GA on Isoproterenol-Induced Inflammatory and Fibrotic Gene Expression Profile in Heart Tissue

**FIGURE 3:**
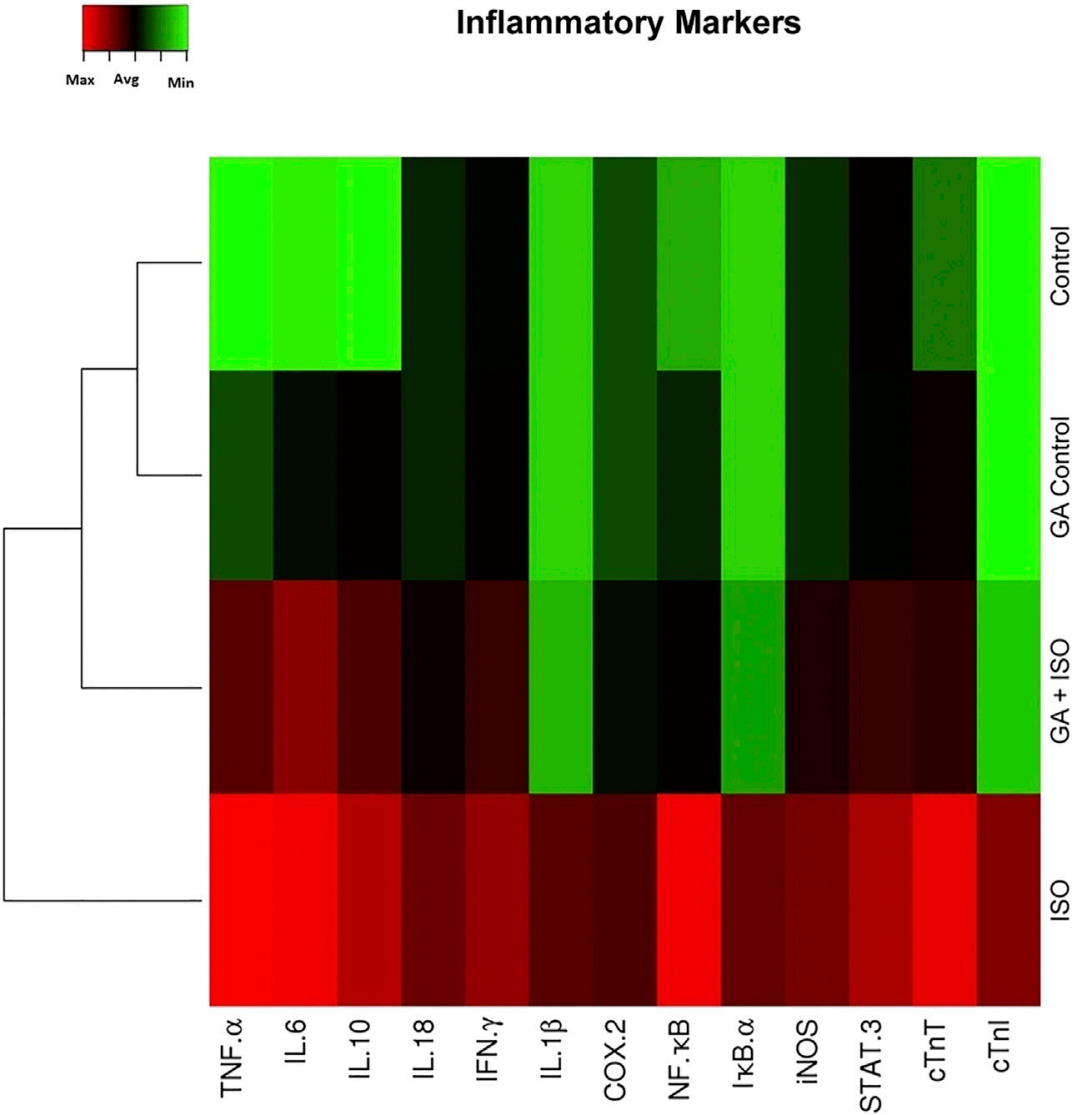
Effect of GA on ISO-induced inflammatory gene expression in heart tissue. Hierarchical clustergram analysis of PCR array results of GA and ISO-induced rats. Bright red indicates the highest normalized signal values, bright green represents the lowest signal values and black represents median signal values. The total mRNA was isolated from heart tissues and were detected using custom PCR array following the manufacturer’s instructions. The clustergram results of three independent experiments were analyzed using the SA Biosciences online tool. Tumour necrosis factor-α (TNF-α), Interleukin-6, -10, -18, -1β (IL-6 IL-10, IL-18, IL-1β), Interferon-γ (IFN-γ), Cyclooxygenase-2 (COX-2), Nuclear Factor kappa-light-chain-enhancer of activated B (NF-κB), Inhibitor of IκB-α (IκB-α), Inducible nitric oxide synthase (iNOS), Signal transducer and activator of transcription-3 (STAT-3), cardiac troponin T and I (cTnT and cTnI).

Generation of inflammatory response is a critical factor in CVDs. The present study showed that the inflammatory genes like TNF-α, IL-6 IL-10, IL-18, IFN-γ, IL-1β, COX-2, NF-κB, IκB-α, iNOS, STAT-3 and cardiac specific genes such as cTnT and cTnI were upregulated in ISO-induced rats (Group III; Figure 3). Pretreatment with GA (Group IV) markedly decreased the levels of both these cytokines and cardiac specific markers.

**FIGURE 4:**
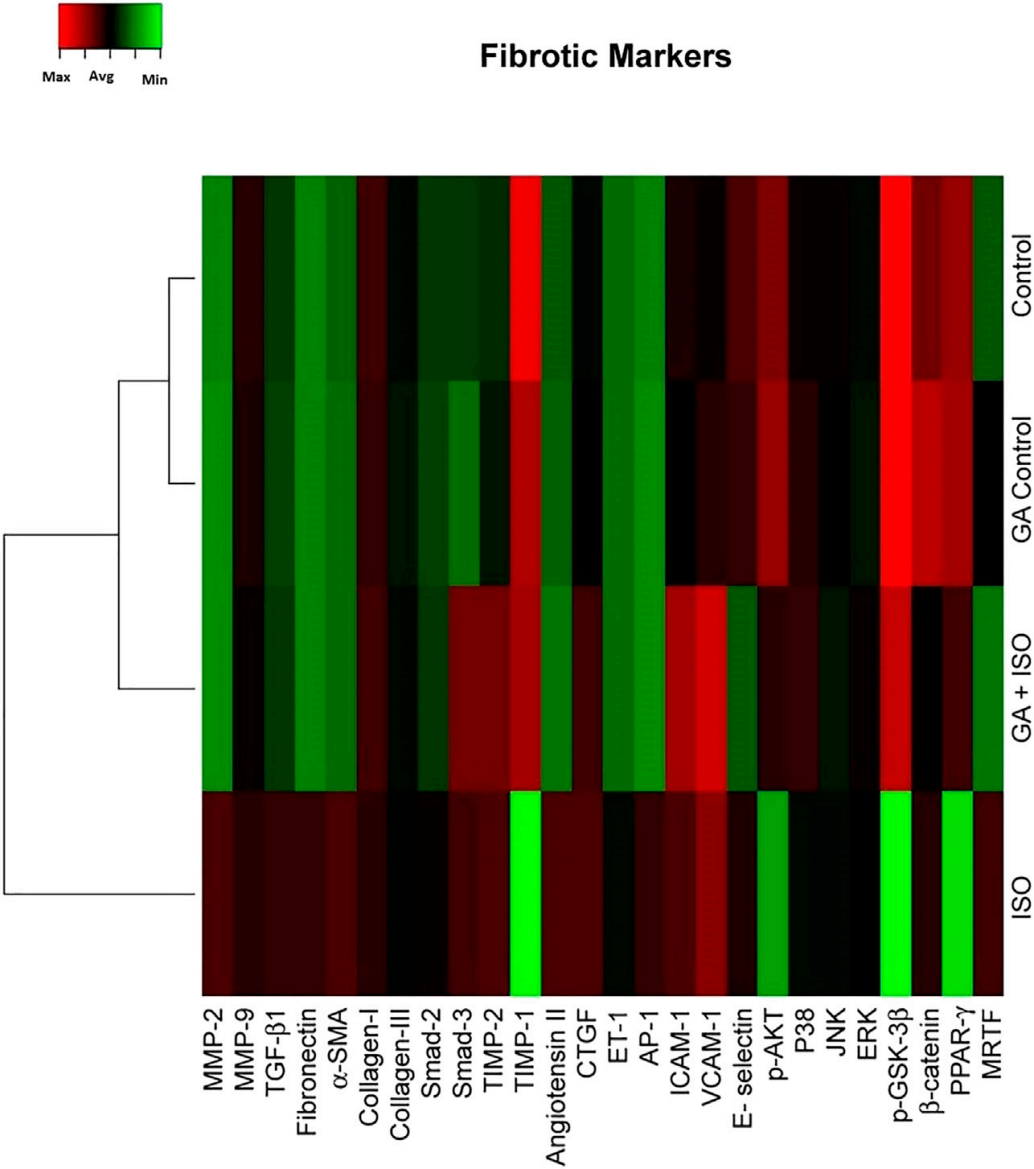
Effect of GA on ISO-induced fibrotic gene expression profile in heart tissue. Hierarchical clustergram analysis of PCR array results for fibrotic gene expression in GA and ISO-induced rats. Bright red indicates the highest normalized signal values, bright green represents the lowest signal values and black represents median signal values. The total mRNA was isolated from heart tissues and were detected using custom PCR array following the manufacturer’s instructions. The clustergram results of three independent experiments were analyzed using the SA Biosciences online tool. Matrix metalloproteinases MMP-2, -9 (MMP-2, -9), Transforming growth factor-beta1 (TGF-β1), Fibronectin, Alpha-smooth muscle actin (α-SMA), Collagen-I, Collagen-III, Smad-2, Smad-3, Tissue inhibitor of metalloproteinases-2, -1 (TIMP-2, TIMP-1), Angiotension II receptor, Connective tissue growth factor (CTGF), Endothelin-1 (ET-1), Activated protein (AP-1), intercellular cell adhesion molecule-1 (ICAM-1), vascular cell adhesion molecule-1 (VCAM-I), E-selectin, p-AKT, p38, c-Jun-N-terminal kinase (JNK), extracellular regulated kinase (ERK), Glycogen synthase kinase 3 ( p-GSK-3β), *ß*-catenin, Peroxisome proliferators-activated receptor- *?* (PPAR-γ) and myocardin-related transcription factor (MRTF).

Fibrosis is another integral feature of cardiac insults, characterized by the abnormal expression of extracellular matrix and accumulation of collagen. In the present study we observed that the fibrotic genes like MMP-2, MMP-9, TGF-β1, fibronectin, *a*-SMA, collagen-I, collagen-III, Smad-2, Smad-3, TIMP-2, angiotension II receptor, CTGF, ET-1, AP-1, ICAM-1, VCAM-I, E-selectin, p38, JNK, ERK, *ß*-catenin, PPAR-γ and MRTF were up regulated expression and TIMP-1, p-AKT, p-GSK-3β and PPAR-γ were downregulated in ISO-treated rats (Group III; Figure 4). Whereas, GA (Group IV) attenuated the fibrosis-linked gene expression in ISO-induced rats.

### Effect of GA on Isoproterenol-Induced Inflammatory Marker Expression in Heart Tissue

**FIGURE 5:**
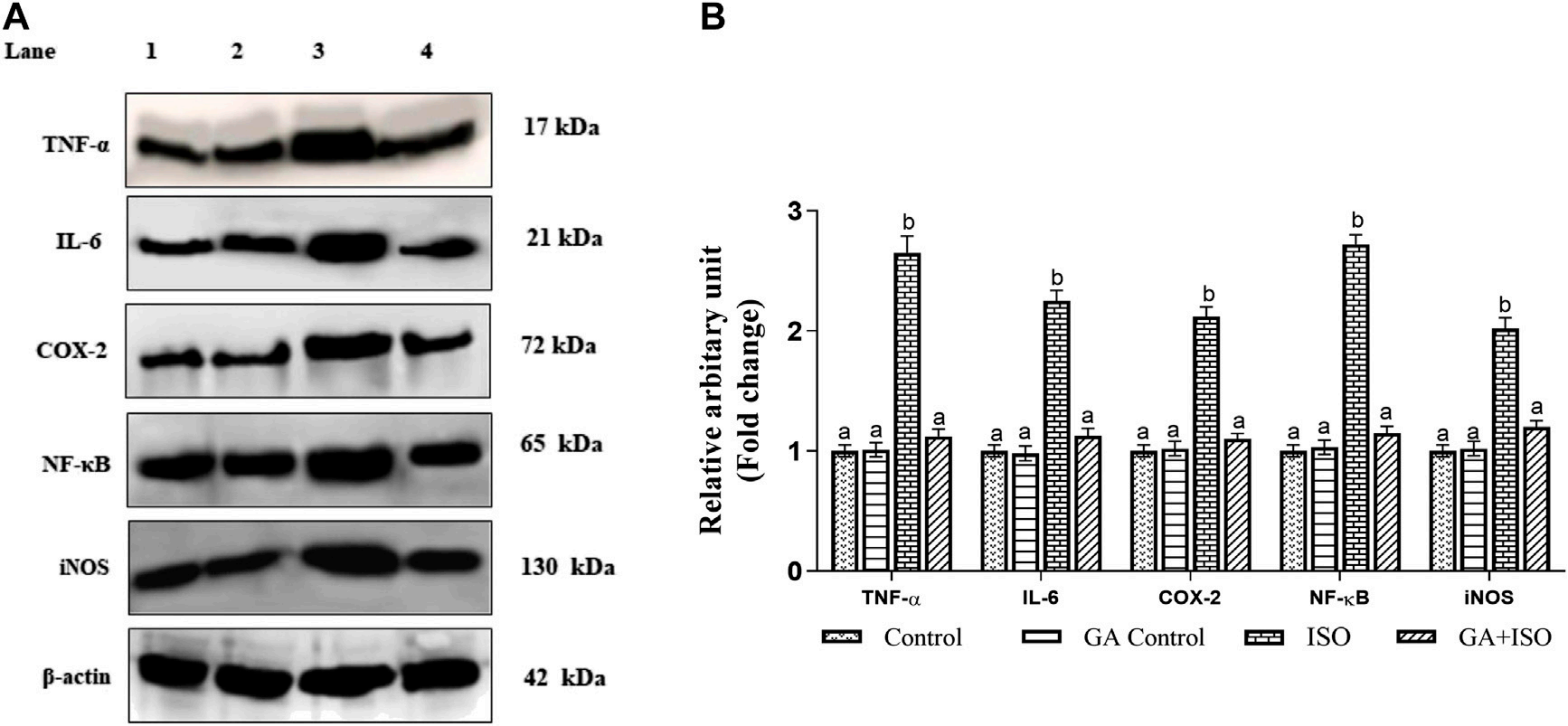
Effect of GA on TNF-α, IL-6, NF-ҡB, COX-2 and iNOS expression in the heart tissue of control and ISO-treated rats. **(A)** Western blot was carried out for the analysis of TNF-α, IL-6, NF-ҡB, COX-2 and iNOS expression and images were acquired by LI-COR using chemiluminescence substrate. Lane 1. Control; Lane 2. GA control; Lane 3. ISO Control; Lane 4. GA + ISO. **(B)** The quantification of protein was performed by densitometric analysis using Image-studio software (LI-COR, United States). The densitometry data represent means ± SD from three immunoblots and are shown as the relative density of protein bands normalized to *ß*-actin. Values not sharing a common superscript (a, b, **C**.) and differ significantly at *p* < 0.05 (DMRT).

The expression of TNF-α, IL-6, NF-ҡB, COX-2, and iNOS was significantly upregulated in ISO-treated rats (Group III). Whereas, GA (Group IV) pretreatment significantly prevented ISO-induced overexpression of TNF-α, IL-6, NF-ҡB, COX-2 and iNOS in rat cardiac tissue (Figure 5A,B).

### Effect of GA on Isoproterenol-Induced Cardiac Fibrotic Marker Expressions in Heart Tissue

**FIGURE 6:**
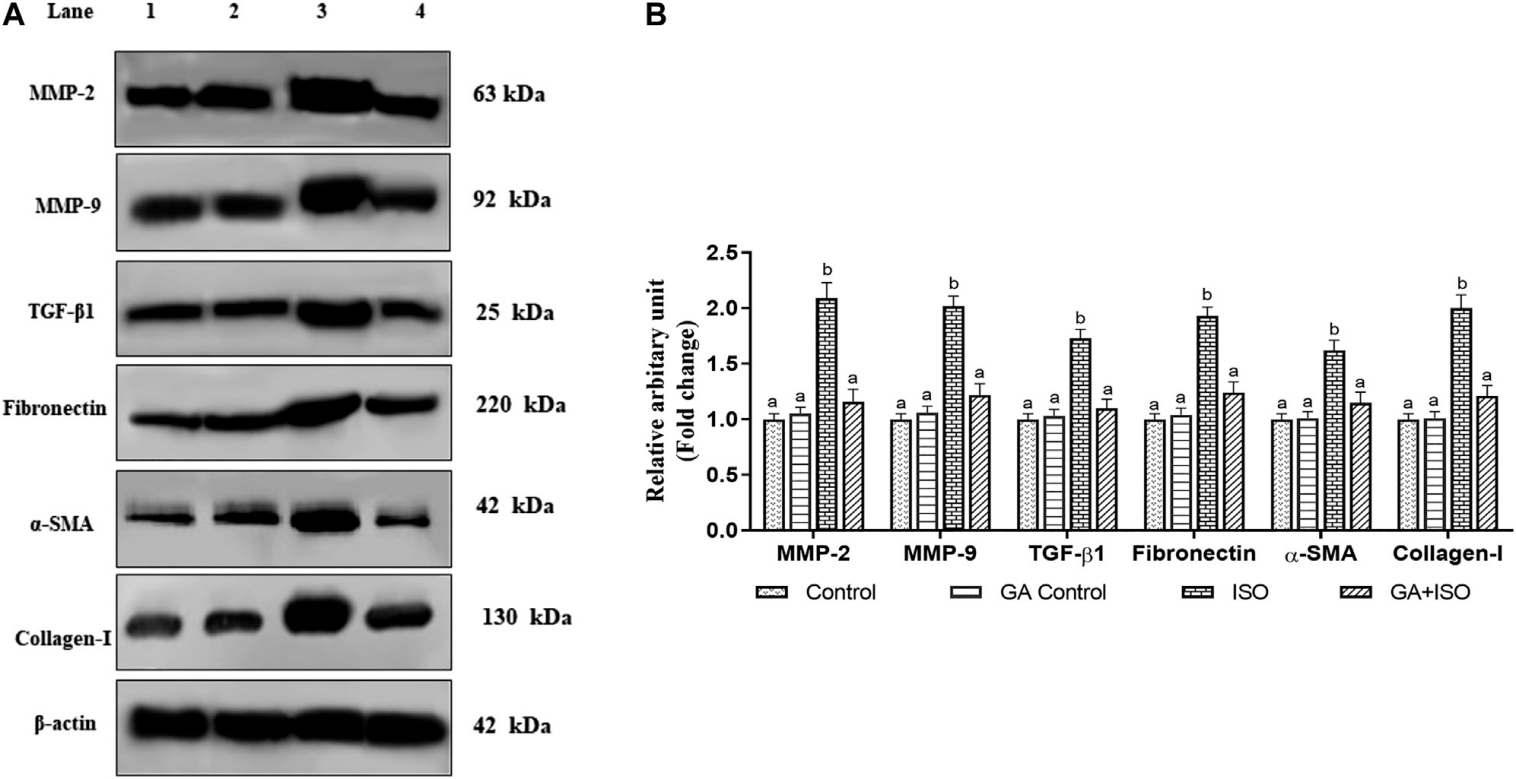
Effect of GA on MMP-2, MMP-9, TGF-β1, fibronectin, *a*-SMA and Collagen-I protein expression in the heart tissue of control and ISO-treated rats. **(A)** Western blot was carried out for the analysis of MMP-2, MMP-9, TGF-β1, Fibronectin, *a*-SMA, and Collagen-I expression and the images were acquired by LI-COR using chemiluminescence substrate. Lane 1. Control; Lane 2. GA control; Lane 3. ISO Control; Lane 4. GA + ISO. **(B)** The quantification of protein was performed by densitometric analysis using Image-studio software (LI-COR, United States). The densitometry data represent means ± SD from three immunoblots are shown as the relative density of protein bands normalized to *ß*-actin. Values not sharing a common superscript (a,b,c…differ significantly at *p* < 0.05 (DMRT).

The cardiac fibrotic markers like MMP-2, MMP-9, TGF-β1, fibronectin, *a*-SMA, and collagen I was examined by western blot analysis. ISO-induced rats (Group III) enhanced the expression of MMP-2, MMP-9, TGF-β, fibronectin, *a*-SMA, and collagen I when compared to control rats. Whereas, GA (Group IV) significantly prevented the expression of MMP-2, MMP-9, TGF-β1, Fibronectin, *a*-SMA, and collagen-I in ISO-treated rats (Figures 6A,B).

### Effect of GA on ISO-Induced Histopathological Changes in Heart Tissue

**FIGURE 7:**
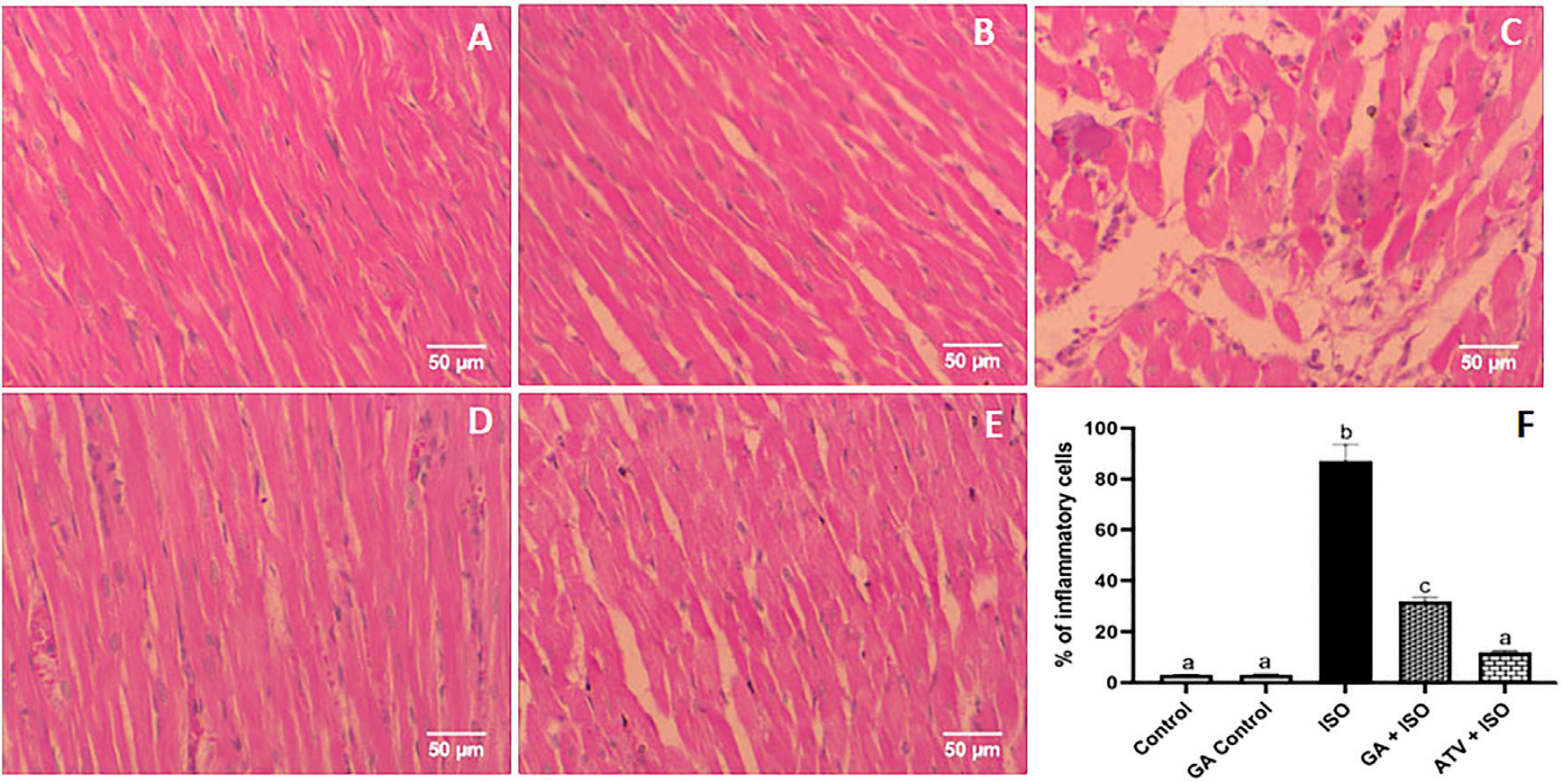
Histopathology of heart tissue stained with H & E staining (40×). **(A)** Control rat shows normal cardiac fibers **(B)**. GA alone treated rats show normal cardiac fibers. **(C)** ISO-induced rat shows degeneration of myocardial fibers, edema, and cellular infiltration. **(D)** Pretreatment with GA showed the decreased degenerated myocardial fibers and cellular infiltrations. **(E)** Treatment with ATV showed normal architecture of cardiac fibers. **(F)** Effect of GA on quantification of inflammatory cells in control and ISO-induced rats by Image J software.

Control and GA-alone treated rats showed normal cardiac fibers (Figures 7A,B). ISO-induced rats (Group III) showed degeneration of myocardial fibers, edema and cellular infiltration (Figure 7C). Whereas, GA pretreatment (Group IV) showed the decrease of the degenerated myocardial fibers and cellular infiltrations (Figure 7D). Treatment with ATV (Group V) showed normal architecture of cardiac fibers (Figure 7E).

**FIGURE 8:**
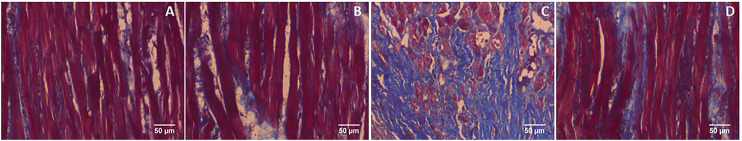
Histopathology of heart tissue stained with Masson's trichrome (40×). **(A)** Control rat shows a normal distribution of collagen. **(B)** GA alone treated rat shows a normal distribution of collagen. **(C)** ISO-control rat shows interstitial collagen accumulation (blue colour). **(D)** Pretreatment with GA showed reduced interstitial collagen accumulation.

Furthermore, GA prevents the cardiac fibrosis as determined by Masson’s trichrome and Picrosirius red staining. Massion trichrome staining showed a normal distribution of collagen in control and GA alone treated rats (Figures 8A,B). ISO-induced rats (Group III) showed interstitial collagen accumulation in the myocardium (blue colour, Figure 8C). Administration of GA prevented ISO-induced (Group IV) interstitial collagen accumulation (Figure 8D).

**FIGURE 9:**
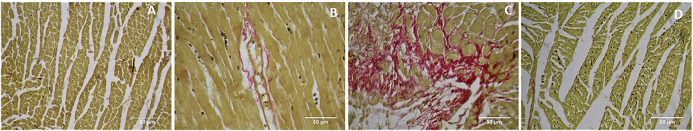
Histopathology of heart tissue stained with Picrosirius red (40×). **(A)** Control rat shows the normal distribution of collagen. **(B)** GA alone treated rat shows the normal distribution of collagen. **(C)** ISO-control rat shows endocardial collagen accumulation (red colour). **(D)** Pretreatment with GA showed reduced collagen deposition in the myocardium.

Picrosirius red staining showed a normal distribution of collagen in control and GA alone treated myocardium (Figures 9A,B). ISO-treatment (Group III) caused endocardial collagen accumulation (red colour; Figure 9C). Pretreatment of GA prevented ISO-induced (Group IV) endocardial collagen accumulation in the myocardium (Figure 9D).

## Discussion

Myocardial infarction (MI) has been considered as a major reason for the chronic heart failure. Experimentally, ISO treatment altered the hemodynamics of heart rate. Dietary phytochemicals have recently been proved as innovative method to help maintain hemodynamics of heart rate and to reduce cardiovascular risk factors. In this study, we observed and a prominent decrease in the systolic and diastolic blood pressures and increased in heart rate in the experimental animals. Improved hemodynamics function by GA/ATV pretreatment clearly indicates its favorable effect on heart in course of the ischemic insult caused by ISO. Several reports illustrates the beneficial effect of phytochemicals on cardiac tissue remodeling (Romão et al., 2019; Komici et al., 2020). Indeed, a large number of experimental models report that phytochemicals maintains homodynamic function of heart rate and significantly prevents heart failure (Paulino et al., 2019; Beshel et al., 2019). During the MI development the cardiac membrane becomes damaged which has resulted in the outflow of cardiac tissue enzymes to the blood stream. Measurement of cardiac tissue enzymes have an important role in the early diagnosis of acute ischemia (Mythili and Malathi, 2015). In this study, we observed that there was an elevated activity of AST, ALT, LDH, CK, CK-MB, cTnT and cTnI in the serum of ISO-treated rats. During the ISO treatment, the cells might get injured due to incomplete oxygen and glucose supply which resulted in the elevated serum level of cardiac marker enzymes (Derbali et al., 2015). Further, we noticed that significant levels of cardiac membrane damages as evident by the increased presence of lipid peroxidation products. ISO-mediated ROS generation might be the reason for elevated cardiac markers, oxidative damages and lipid peroxidation (Montezano et al., 2015). Our results clearly illustrates that GA treatment prevented the levels of ISO-induced cardiac markers in the blood stream. This cardioprotection property of GA might be due to its inherent antioxidant property. The GA possesses several structural motifs which are responsible for its antioxidant potential (Sohn et al., 1998). The presence of C-2–C-3 double bond and a C-3 hydroxyl group in GA has been highlighted for its antioxidant potential. Specifically, the hydroxyl group present at the C3 position of GA exhibit higher antioxidant activity than other similar flavonoid apigenin which bears a hydroxyl group at C4 (Gregoris and Stevanato, 2010). Free radicals like superoxide and singlet oxygen have already shown to be scavenged by GA (Aloud et al., 2017). Further, GA has been reported to improve cellular antioxidant status and reduces oxidative stress related damages in diabetic rats (Zeng et al., 2019). There are few redox sensitive pathways are activated during the development of MI. The ISO treatment induced overexpression of inflammatory cytokines and NF-kB p65 transcription factor in the experimental animals which resulted in heart failure (Hamid et al., 2011). The present study revealed that ISO-treated rats showed significantly upregulated expression of NF-κB and subsequent overexpression of pro-inflammatory cytokines in the heart tissue. Interestingly, GA treatment prevented ISO-induced inflammatory responses in the myocardial tissue. The anti-inflammatory role of GA has also been proved in other inflammatory experimental models. For example, GA against lipopolysaccharide-activated macrophages via NF-κB pathway regulation (Wang and Tang, 2017). Choi et al. showed the anti-inflammatory mechanism of GA in LPS-mediated inflammation through PPAR-γ signaling (Choi et al., 2017). GA shows anti-inflammatory effects by affecting gene expression. This could be attributed to the fact that flavones and hydroxyflavones can inhibit the phosphorylation of proteins involved in the signal transduction including regulation of NF-κB (Torre, 2017). It is possible that GA might block the activity of IκK, suppress IκB degradation and prevent the activation of NF-κB thereby regulating the suppression of IL-6 and TNF-α production. GA has been proved to inhibit inflammatory via negative regulation of NF-κB in renal tissue (Lu et al., 2019). The anti-inflammatory effect of GA and similar flavonoids has also been proved in LPS-stimulated RAW 364.7 macrophages and DNCB-mediated dermatitis in experimental models (Lee et al., 2018).

The MMP-2 and MMP-9 are the key players of myocardial fibrosis which have been found elevated during ISO-treatment. Conversely, GA significantly decreased the ISO-mediated expression of MMP-2 and -9 thereby contribute to the attenuation of cardiac fibrosis. The modulatory role of GA on NF-κB signaling could be correlated for its effect on MMPs expression and cardiac fibrosis. Yang et al. showed the inhibitory potential of GA against thrombin-induced MMP-9 expression in SK-N-SH cells via protein kinase-dependent NF-κB phosphorylation (Yang et al., 2018). It has also been reported that GA treatment attenuated the expression TGF-β1/Smad signaling induced pressure overload (Liu et al., 2015).

Cardiac collagen tissue remodeling is an important event in the progression of heart failure. Excessive accumulation of the matrix fibrillar collagens is the major hallmark of myocardial fibrosis. This event has been considered as a key feature of cardiomyopathies which compromises cardiac systolic and diastolic performance (Takawale et al., 2017). Matrix metalloproteinases (MMPs) and their inhibitors, tissue inhibitor of metalloproteinases (TIMPs), directly impact the ECM turnover and homeostasis (Fan et al., 2012). Apart from MMPs, several molecules that include fibronectin, *a*-SMA, TGF-β1 etc has been involved in the pathogenesis of myocardial fibrosis. Further, tissue inhibitors of metalloproteinases (TIMPs) has also play an important role in the management of cardiac tissue architecture (Li et al., 2000). The abnormal production of tissue inhibitor of matrix metalloproteinases type 2 (TIMP2) might repair myocardial functions which resulted in cardiac hypertrophy (Fan et al., 2019). In this study, we observed that GA pretreatment prevents ISO-induced cardiac fibrosis by attenuating collagen accumulation and by downregulating the expression pattern of MMP-2, MMP-9, TGF-β1, fibronectin, *a*-SMA, collagen type I, III, Smad-2 and -3. In addition, GA markedly elevated the expression of TIMP-1 and attenuates the TIMP-2 expression in ISO-induced rats.

The cardiac fibroblast is a remarkably versatile cell type that coordinates inflammatory, fibrotic and hypertrophic responses in the heart through a complex array of intracellular and intercellular signaling mechanisms. One important signaling mode that has been identified involves p38 MAPK; a family of kinases activated in response to stress and inflammatory stimuli that modulates multiple aspects of cardiac fibroblast function, including inflammatory responses, myofibroblast differentiation, extracellular matrix turnover and the paracrine induction of cardiomyocyte hypertrophy (Turner and Blythe, 2019). In this study, GA treatment inhibited the activation of JNK, ERK and p38 in ISO-induced rats. Similarly, GA treatment attenuated the expression of p38, ERK and JNK in LPS-induced RAW264.7 cells (Lee et al., 2018). It was also found that PPAR-γ activation has also been blocked by GA pretreatment. Myocardin-related transcription factor (MRTF)/serum regulatory protein (SRF) is also an important transcription regulator involved in myocardial fibrosis. In this study, GA modulates the ISO induced activation of MRTF/SRF and this could be due to regulation of other redox-sensitive transcription factors like NF-κB and MAPK signaling molecules. Liao et al. reported that myocardin expression is induced in cardiac hypertrophy, and its overexpression in neonatal rat cardiomyocytes induces hypertrophy (Liao et al., 2011). GA significantly ameliorated cardiac function and inhibited cardiac hypertrophy and fibrosis by disruption of PI3K–Akt–GSK3β and MEK1/2–ERK1/2– GATA4 signaling and via Ang II stimulation (Wang et al., 2013; Wang et al., 2019). According to the present findings, GA plays an important protective role in regulating cardiac fibrosis. Histological observations showed that GA prevented ISO-induced degeneration of myocardial fibers, edema, and cellular infiltration. Masson’s trichrome staining also illustrates GA prevented ISO-induced interstitial collagen accumulation in the myocardium (blue colour). Picrosirius red staining further substantiates the preventive effect of GA against ISO-induced endocardial collagen accumulation (red colour) in rat myocardium.

## Conclusion

The present results suggest that GA exhibits strong cardioprotective effects against ISO-induced myocardial infarction in rats. The protective effect of GA was observed by preventing ISO-induced depletion of endogenous antioxidants and cardiac marker enzymes. Additionally, GA prevented ISO-induced inflammatory signaling and fibrotic pathway in the heart tissue. These findings provide a potential application of GA in the prevention and treatment of cardiac fibrosis.

## Data Availability Statement

The raw data supporting the conclusions of this article will be made available by the authors.

## Ethics Statement

The animal study was reviewed and approved by The Institutional Animal Ethical Committee, Annamalai University.

## Author Contributions

We declare that this research work was done by the authors named in this article. Study concept: NRP. Study design: NRP and GK. *In vivo* experiments: TR and AS. Statistical analysis and interpretation: NP and TR. Manuscript preparation: NRP and TR. Manuscript editing: NP, GK, HK, and AA. Funding acquisition: HK and AA. All authors contributed to the article and approved the submitted version.

## Funding

The financial support to T. Radhiga, Postdoctoral fellowship scheme (File No. F./PDFSS-2014-15-SC-TAM-9339 dated 03.02.2015) from University Grants Commission, New Delhi, is gratefully acknowledged. The authors would like to extend their sincere appreciation to the Deanship of Scientific Research at King Saud University for funding the Research Group No. RGP-009.

## Conflict of Interest

The authors declare that the research was conducted in the absence of any commercial or financial relationships that could be construed as a potential conflict of interest.
